# Using person-specific networks in psychotherapy: challenges, limitations, and how we could use them anyway

**DOI:** 10.1186/s12916-020-01818-0

**Published:** 2020-11-23

**Authors:** Lino von Klipstein, Harriëtte Riese, Date C. van der Veen, Michelle N. Servaas, Robert A. Schoevers

**Affiliations:** University of Groningen, University Medical Center Groningen, Department of Psychiatry, Interdisciplinary Center Psychopathology and Emotion regulation (ICPE), PO Box 30.001 (CC72), 9700 RB Groningen, The Netherlands

**Keywords:** Person-specific networks, Case conceptualization, Experience sampling method, Idiographic models

## Abstract

**Background:**

The complexity of psychopathology is evident from its multifactorial etiology and diversity of symptom profiles and hampers effective treatment. In psychotherapy, therapists approach this complexity by using case conceptualization. During this process, patients and therapists closely collaborate on a personalized working theory of the patient’s psychopathology. This is a challenging process and shows low reliability between therapists. With the experience sampling method (ESM), time-series data—valuable for case conceptualization—can be systematically gathered in a patient’s normal daily life. These data can be analyzed and visualized in person-specific networks (PSNs). PSNs may support case conceptualization by providing a schematic representation of association patterns between affective, cognitive, behavioral, and context variables.

**Main text:**

We adopt a clinical perspective in considering how PSNs might be implemented to serve case conceptualization and what their role could be in psychotherapy. We suggest PSNs to be based on personalized ESM assessment to capture the unique constellation of variables in each patient. We reflect on the lack of a gold standard for creating PSNs, which may result in substantially different PSNs and thereby disparate information for case conceptualization. Moreover, even if PSNs are created in a consistent manner, results remain ambiguous as they are subject to multiple interpretations. Therefore, associations in PSNs do not allow for firm conclusions about a patient’s psychopathology, but they may nevertheless be valuable in the process of case conceptualization. PSNs are based on systematically gathered, ecologically valid ESM data and provide a unique personalized perspective. When used responsibly, PSNs may be able to support case conceptualization by generating questions that serve as a starting point for a dialog between therapists and patients. Well-targeted questions are an essential tool for therapists to gain insight into the patients’ psychopathology patterns and improve the quality of case conceptualization.

**Conclusions:**

PSNs have limitations in terms of the reliability of the insights they provide directly. However, taking these challenges into account, we believe they have potential as a tool to help therapists and patients in their collaborative exploration of a patient’s psychopathology. Clearly, this would need to be validated in future clinical research.

## Background: the appeal of person-specific networks

In the effort to better understand psychopathology and help those suffering from it, it is a great challenge to deal with its inherent heterogeneity. Patients with the same diagnosis may show different symptom profiles [1], which may also vary over time. Moreover, the same symptoms may be present in different diagnoses and are not distinctive for one single disorder [2, 3]. Furthermore, studies consistently show high comorbidity between mental disorders [4–6]. In line with its symptomatology, the etiology of mental disorders also is highly multifactorial and largely unspecific [7, 8]. This diversity is not adequately captured by diagnostic disorder categories or the associated disease model, which holds that a given pathology can be explained by a single underlying cause [9]. It is not surprising then that protocolized treatments for specific forms of psychopathology are only effective for a portion of patients [10]. Clearly, the question “what works for whom” is crucial for the field, and it seems desirable that we complement current diagnostic systems with tools that provide more individualized insights in order to support intervention selection.

While large and complex studies are needed to approach this question on a scientific level [11], therapists have to answer this question in their daily work. One important method therapists use to deal with this question is case conceptualization (CC; or case formulation). In CC, therapists and patients develop a working theory by collaboratively integrating information from (i) the patient’s background, current situation, and introspections and (ii) the therapist’s experience and theoretical knowledge. The goal is to create a theory that captures the patient’s unique constellation of relationships between emotions, behaviors, cognitions, somatic states, and context, so it can be used to guide personalized treatment decisions. However, CC is a challenging process, as it involves complex problem solving [12] without a gold standard procedure [13]. Moreover, CC is based on information that could be incomplete or biased, because it depends on the type of questions being asked and the retrospection of the patient. Accordingly, studies show low reliability of CC between therapists [14, 15].

**Fig. 1:**
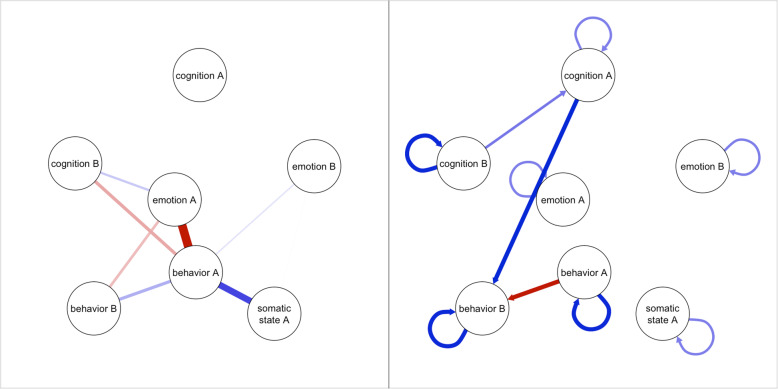
Two examples of person-specific network graphs. Circles represent variables (e.g., emotion, cognition), and lines between circles represent associations. The color of lines indicates whether an association is positive (blue) or negative (red), and their thickness and transparency indicate their relative strength. The left panel shows a contemporaneous network with undirected connections, which represent the partial associations between the two variables measured at the same time point. The right panel shows a temporal network with directed connections, which indicate that a variable at time point *t-1* (origin of arrow) is partially associated with a variable at time point *t* (point of arrow)

Recent developments in the field provide tools that may address this challenge. The experience sampling method (ESM) assesses momentary states during normal daily life through short questionnaires that are typically filled out multiple times per day on a smartphone [16]. The resulting time-series data have high ecological validity and are less affected by retrospective bias. Interestingly, ESM time-series data can be analyzed with different statistical models that may reveal patterns in daily life dynamics of individual patients [17, 18] and thus provide a useful source of information for CC. One category of such person-specific (or idiographic) statistical models is psychological network models [19], which were inspired by the broader framework of the network approach to psychopathology [20]. Person-specific psychological network models can be used to analyze associations between a set of variables in a particular patient. These models can include two types of associations: contemporaneous associations (i.e., symptom A is associated with symptom B at the same time point) and temporal associations (or lagged associations, i.e., symptom A is associated with symptom B at the following time point). The estimated associations can be visualized in a person-specific network^1^ (PSN) graph to facilitate interpretation by the therapist and patient (see Fig. 1 for an example).

The intuitive appeal of PSNs in the context of CC is that they may provide information on how elements of the individual patient’s psychopathology are functionally related a core task in CC. This appeal is especially strong in CC for cognitive behavioral therapy (CBT), where models of psychopathology are strongly based on the situational functional relationships between cognitions, emotions, and behaviors [21]. If PSNs can substantially support CC in understanding such relationships, they would be of considerable clinical value. Some researchers have even suggested that PSNs may directly indicate treatment targets [22, 23]. Several initial case studies, which collected data through ESM and created PSNs for individual patients, have shown that PSNs were feasible, well-received by patients, and provided clinically relevant information [24–27]. These case studies give the first indication that PSNs may indeed provide added value for CC. However, there are also findings that therapists report doubts on whether PSNs can provide new information [26, 28].

While the concept and results from some initial case studies of PSNs are promising, their implementation in clinical practice involves many choices and a balanced consideration of clinical, methodological, and practical issues. In this article, we attempt to navigate these issues in order to provide an overview of the choices and considerations involved and to get a more concrete idea of the clinical potential of PSNs. We focus particularly on clinical considerations, which have been underrepresented in the literature thus far. First, we consider how we can create PSNs for the goal of supporting CC. Second, we consider what a PSN can and cannot tell us about an individual patient. Finally, we reflect on the potential clinical value of PSNs, their potential role in the process of CC, and how this may inform future research. Our considerations were informed by our implementation of PSNs in an ongoing randomized control trial—the *Therap-i* study.^2^ This trial implements PSNs as part of a larger intervention using personalized ESM to support CC in psychological treatment for depression, see Table S1 (Additional file 1) for the choices we made in data collection, analysis, and visualization to create PSNs in the *Therap-i* study.

## How to create person-specific networks—a clinical perspective

In light of the inherent heterogeneity in psychopathology, we strongly suggest that PSNs are based on data measured through personalized ESM questionnaires. We also suggest that these questionnaires are created in close collaboration between patient and therapist. Such collaboration is a key principle of CC [29], as it makes use of the introspective capability of the patient and the clinical expertise of the therapist. A recent study showed that patients and therapists both recommended the collaborative personalization of the ESM questionnaire for the implementation of ESM in clinical practice [30]. Personalized ESM has not been empirically studied yet but is currently being investigated in the *Therap-i* trial. In order to create a personalized ESM questionnaire for CC in clinical practice, patients and therapists need to first identify the factors that they deem (potentially) important for explaining the patient’s psychopathology. Importantly, this also includes the identification of the strengths and adaptive skills of the patient, an area that is central to CC, but can be underrepresented in clinical practice because of the primary focus on psychiatric complaints [29]. Like in CC, factors may stem from a variety of sources: the patient’s background, current situation, and introspections, as well as the therapist’s experience, theoretical knowledge, and appraisal of the case. To structure the collection of factors, we suggest that patients and therapists organize factors into five domains that cover important aspects of CC: affect, behavior, cognition, somatic states, and context. This may also help them with creating an ESM questionnaire that covers all relevant domains of CC. Once factors are identified, patients and therapists need to select fitting ESM questions that capture the expression of the factors in the daily life of the patient. They can be enabled to do this independently by applications developed to implement ESM in clinical practice [31].^3^ Such an application needs to be user-friendly and guide patients and therapists in the process, since they have limited time and experience with ESM compared to researchers. A key component could be an organized repository of ESM questions that provides a range of ESM questions that patients and therapists may select from. Additionally, therapists could be trained to formulate ESM questions themselves, in case the repository does not include a fitting question. Formulating questions in the patient’s own words may also increase their ability to answer accurately and increase compliance [32]. Besides the personalized questions, we suggest that questionnaires also contain some fixed questions on aspects of everyday life that are relevant for everyone (e.g., questions on affect, social interactions, daily events).

Creating a personalized ESM questionnaire in this way is in itself an act of starting CC. Accordingly, it can collect a considerable number of ESM questions. Pilots and first experiences in the *Therap-i* trial have shown that personalized ESM questionnaires for CC typically contained 20–30 questions. Studies that apply (non-personalized) ESM questionnaires in clinical samples, including those from our own group, have used a similar or higher number of questions [32–35], and compliance rates suggest a manageable burden for participants. Such numbers are at odds with statistical considerations. From a statistical perspective, the number of variables should not exceed a certain maximum to ensure sufficient power. With low statistical power, relevant associations may not be found in PSN modeling. The maximum number of variables that are adequate from a statistical perspective depends on a number of circumstances, and clear recommendations are not available at the moment. However, given that the amount of data that can be collected in a clinical setting is limited, this number is likely less than 10.^4^ The difference between this and the number of (single-question) variables in an ESM questionnaire illustrates that creating PSNs clearly requires difficult compromises between comprehensive ESM assessment and sound statistical modeling. For further considerations on ESM measurement, we refer interested readers to the ESM literature [32, 36–39], as these are not specific to personalized ESM in the context of PSNs per se.

Once data are collected, a number of analysis and visualization steps follow to ultimately create a PSN. Large-scale application in clinical practice requires that these steps follow a standardized procedure, enabling the automated generation of PSNs. This is also necessary to be able to research the effectiveness of using PSNs in psychotherapy. However, deciding on a standardized procedure is difficult since there is no gold standard approach for many of the steps involved. This is illustrated by the fact that proof-of-principle studies applying PSNs have made different choices in creating PSNs [24–27]. Currently, there are many possible paths to create PSNs, and we often do not know how to choose between them. This is problematic because the resulting PSNs may differ substantially [40]. Bastiaansen and colleagues [41] provided an elegant illustration of this problem. They asked different research teams to recommend treatment targets based on the same ESM time-series data of one particular patient. Most research teams included a PSN in their analysis, but choices in the statistical analyses and recommendations for treatment varied widely. Further methodological research is necessary to arrive at clearer recommendations on how to create PSNs for use in clinical practice.

## The interpretation of person-specific networks

Once a PSN is created, it has to be interpreted in order to serve as a tool to support CC. Assuming that we are confident in the collected ESM data and the choices made in modeling and visualizing PSNs, two major points are important with regard to their interpretation.

### Person-specific networks show part of the picture

If PSNs are used as a tool to support CC, it is important to recognize that they rely on simplifications and assumptions, which are inherent to the measurement and modeling methods they are based on. Accordingly, they can capture some, but not all, processes. First, PSNs can capture aspects that are captured in the ESM questionnaire. On the one hand, that means that some aspects are not captured. Some aspects may simply be missed when the questions for the ESM questionnaire are chosen. Further, there are certain aspects of CC that cannot be adequately captured on the rating scale of a questionnaire because some aspects are inherently qualitative in nature. Most prominently, the diversity of context (i.e., the situations and environments a patient encounters) can only be captured as far as it can be reduced to a set of quantitative variables. Similarly, a patient’s upbringing and past life events, which provide important background information for CC, are only considered as far as they are traceable in momentary quantitative variables (e.g., cognitions). On the other hand, ESM is able to capture variables of central interest to CC, namely momentary expressions of affect, behaviors, cognitions, and somatic states. Also, these aspects are captured with high ecological validity and are less affected by retrospective bias [42]. Second, PSNs capture associations on two specific time scales, namely contemporaneous associations between variables measured at the same moment and temporal associations between variables typically measured at consecutive moments. On the one hand, this could be problematic if we consider that the processes we are interested in may take place on heterogeneous time scales and time scales other than the ones analyzed. Especially slower processes that develop over the course of days or longer are not represented, because ESM-based PSNs typically focus on effects over the course of hours. For example, in an unfit person, physical activity may be associated with negative affect on a time scale of hours. However, through a training effect, physical activity might foster more positive affect over days or weeks. There are methods to consider associations on multiple time scales [43]. However, such methods further increase the complexity of PSNs, which are already difficult to understand for patients. On the other hand, although limited to two time scales, PSNs are able to capture many processes interesting to CC. PSNs capture processes that occur on short time scales (e.g., in the order of minutes), because such processes are traceable in contemporaneous associations [25]. It seems plausible that many processes between variables in a PSN occur on such a time scale. This assertion is in line with CBT, which involves the analysis of connections between momentary affect, behaviors, cognitions, and somatic states in specific moments [21]. Third, PSNs only capture processes that are dynamic to begin with. That is, there needs to be variation in all included variables. Therefore, phenomena that are infrequent relative to the ESM measurement frequency (e.g., panic attacks) cannot be captured [44]. Fourth, PSNs capture associations that are consistent over time, or whose inconsistency (variation) is captured in the included variables. On the one hand, associations that are inconsistent over time are not adequately captured. One reason that such inconsistency is likely to occur is the fact that patients react differently in different contexts and we cannot adequately include the context in a PSN. Social psychology teaches us how important context is in determining how people feel, think, and behave [45]. Another reason to expect inconsistency in associations is the complex dynamic systems theory, which suggests that psychological processes are perpetually changing [46]. On the other hand, it is also worth considering that processes that are consistent and independent of context might be the more important and pathological ones. Fifth, PSNs assume that the mean of included variables is stable over time and only capture associations between momentary deviations from that stable mean. Changes in the mean are considered an artifact to correct for. However, such changes in the mean are potentially more meaningful than momentary changes that dissipate quickly—they may provide information about what may drive lasting change in the patient and are the very goal of psychotherapy. Sixth, PSNs capture linear associations between variables but may misrepresent associations that are non-linear. These considerations show that not all relevant processes can be captured in PSNs, but what can be captured is considerable and relevant to CC.

### Person-specific networks provide ambiguous information

Under the condition that a causal process is captured by the PSN (see the previous section), causation implies correlation and the process will show as an association in the PSN. However, drawing conclusions about causality on the basis of associations is not warranted because correlation does not imply causation. An association between a pair of variables may be explained by multiple different processes. The direct line between two variables in a network graph appears to suggest a direct (causal) relationship between them, but there are several other possibilities: (i) the relationship could be causal but indirect, mediated through some unobserved variable; (ii) it could also be explained by an unobserved variable that affects both variables (a common cause); and (iii) it might even be spurious, if the two variables both have an effect on a variable that is included in the network (a so-called collider structure) [47]. Thus, it remains ambiguous what process may have led to an association found in a PSN. Beyond this ambiguity, special care is warranted in interpreting temporal associations. Although they may fulfill the criteria for Granger causality, they are not evidence of causality [48]. Consider, for example, a socially anxious person, who experiences anxiety in anticipation of social interactions. While anxiety would temporally predict social interaction, causality operates in the reverse direction: the anticipation of social interactions causes anxiety. In summary, while associations in a PSN contain the signals of causal processes, they remain ambiguous. Therefore, it is important to firmly stick to a correlational interpretation of PSNs.^5^

## Using person-specific networks in psychotherapy

Taking together these considerations, it is clear that PSNs cannot directly provide reliable insights into a patient’s patterns of psychopathology nor recommendations on treatment targets. Although some researchers have proposed that PSNs could yield such firm conclusions [22, 23], the use of PSNs in psychotherapy has mostly been framed as exploratory [25, 44]—providing suggestions rather than answers. Such exploratory framing allows for the idea that PSNs can be useful without being entirely fail-safe. An association found in a PSN could be both a clue to an important mechanism or a misleading artifact. Given an exploratory use of PSNs, two central questions arise: First, is there a responsible way to implement PSNs in psychotherapy, utilizing its benefits without misleading patients and therapists? Second, given that there is a way, how valuable are suggestions from PSNs for psychotherapy?

### Using person-specific networks responsibly

Given the abovementioned limitations in interpreting PSNs, patients and therapists are likely to over-interpret PSNs in clinical practice. The collection of “objective” data, use of statistical modeling, and smooth visualizations create an appearance of objectivity that is not supported by firm evidence. With this in mind, the framing that PSNs provide suggestions is not very helpful, as it is not instructive on *how* trustworthy a PSN is, which leaves the door open for over-interpretation. We therefore suggest that PSNs are used as a tool to generate questions that may guide further collaborative exploration of a patient’s psychopathology. As is best practice in CC [29], these questions can then be collaboratively explored and discussed between the therapist and patient. Thereby, they are measured against the patient’s introspective capability and the therapist’s clinical expertise. To further facilitate the responsible and productive use of PSNs, we suggest training therapists. Core lessons of therapist training could include the following: how to create a personalized ESM questionnaire, the correlational interpretation of associations in PSNs, “correlation does not imply causation,” “PSNs show part of the picture,” how to communicate the interpretation of PSNs to patients (e.g., through examples), and suggestions on how to explore associations in a PSN. On the latter, we would suggest that therapists follow a correlational interpretation up with “do you recognize this?” and “let us talk about why this occurs, do you have an idea?” Additionally, they might use descriptives of the collected ESM data to further explore associations.

### The potential of person-specific networks

If PSNs are used to generate questions, the clinical value of PSNs in psychotherapy is determined by the added value of these questions. PSN-generated questions can only point to processes that are captured in their associations. As we have argued above, a part of relevant processes is likely captured in PSNs. PSN-generated questions may point to different processes than traditional CC, as they are conceived through a very different process. They provide a novel perspective that may be valuable, because it is difficult to obtain a comprehensive overview and understanding of a patient’s psychopathology. Just like PSNs, patients and therapists may only see part of the picture based on the retrospective information discussed in therapy sessions and are naturally limited by their own perspectives and biases. It is a key principle of CC to question one’s own perspective and to be open to new information [49]. Therapists put this into practice precisely by asking the patient questions, allowing for bottom-up creation of personalized knowledge and insight. Asking good questions is considered a key skill for any therapist and receives considerable attention in their training through techniques like Socratic questioning [50] and motivational interviewing [51]. PSNs may have the most potential when and where there is a need to simulate the collaborative exploration in CC. Natural moments of exploration in psychological treatment are the start of treatment and moments of evaluation and reorientation. Furthermore, additional investment into CC is most indicated in the treatment of patients with complex psychopathology (e.g., with comorbidities). In their case study, Kroeze and colleagues provided an example of a stimulating discussion about a PSN [27]. Their discussion led to the formulation of causal hypotheses and motivated the patient to try a new treatment direction, which she previously had had doubts about.

Besides the direct benefit of questions generated by PSNs, PSNs may also have secondary benefits for psychotherapy that result from the way PSNs are created and presented.^6^ First, ESM assessment itself is increasingly considered an intervention for psychopathology, and there is the first evidence of its benefits [30, 52]. The idea is that regularly answering ESM questions may stimulate awareness, reflection, and insight. Second, when the ESM questionnaire is personalized in collaboration between patient and therapist, this may foster their working alliance. By providing a structured process for this collaboration, it ensures that the perspective of the patient is well represented in CC. Third, the pure concept of networks, which is illustrated by network graphs, may be useful to patients, as it teaches them a framework of psychological thinking, namely the idea of interacting psychological elements [53]. Patients learn that the path towards recovery is not linear (from sick to healthy), but more complex, and that they can learn to influence the larger system by learning to influence single elements. Lastly, the gathered ESM time-series data can easily be presented to patients and therapists descriptively (e.g., by plotting variable course over time). This provides them with an interesting additional source of information that may well be used to further explore new PSN-generated questions. For example, they may identify concrete moments in which two variables peak together, consult text descriptions of the situation collected in the ESM assessment, and explore these moments in depth. This example also illustrates a larger point: The different benefits of PSNs integrate well with each other and form a full CC process.

While we have argued that PSNs have potential despite their limitations, the added value they contribute is ultimately a question for further empirical research. While the question of how many relevant processes are captured by PSNs is essential, it is also very hard to investigate empirically. In contrast, the question of added clinical value can be investigated more readily. Specifically, we suggest that future research investigates (i) whether PSNs generate additional and useful questions whose exploration can inform patients and therapists about the patients’ psychopathology (CC), (ii) whether exploration of such questions provides additional and useful information compared to the information gathered through questions asked during the typical CC process, (iii) whether the idea of networks teaches patients to think of their problems as complex systems, and (iv) whether any benefits outweigh the costs of time invested in training therapists and in discussing PSNs with patients. Also, as PSNs are based on ESM data, research on the benefits and burdens of (personalized) ESM assessment in clinical practice will be essential for judging their added value.

Beyond the topic of PSNs, many of the points and suggestions we make may also generalize to the use of other idiographic models in clinical practice. Ultimately, any idiographic model is bound by its assumptions and based on less-than-perfect data, making far-reaching recommendations problematic. Navigating this in the way we have suggested—using a model to ask questions and stimulate exploration—provides a responsible framework to investigate their innovative potential.

## Conclusion

PSNs in psychotherapy, like most statistical models in psychiatry, impose a structure on a complex reality. This structure may help us navigate the complexity of psychopathology, but because it relies on certain simplifications, it can also point to the wrong direction. Yet, if we imbed PSNs into psychotherapy in a responsible manner, it may prove useful. By using PSNs to guide collaborative exploration, but firmly keeping the responsibility for developing a working theory of the patient’s pathology with patient and therapist, PSNs may prove an important guiding tool in the collaborative CC process.

## Supplementary information

**Additional file 1:** **Table S1.** Procedure for creating person-specific networks in the Therap-i trial.

## Data Availability

Data sharing is not applicable to this article as no datasets were generated or analyzed during the current study.

## Footnotes

1 We use the term person-specific network to refer to the end product that is the result of modeling and visualization.

2 Registered prospectively at the Dutch Trial Register, NTR 7381, 03-08-2018.

3 To meet this challenge, our group is currently developing the PErsonalized Treatment by Real-time Assessment (PETRA) tool.

4 In a scenario with 10 variables, there are 100 temporal and 55 contemporaneous possible association parameters to be estimated. The total number of parameters thereby approximately matches the number of data points that can realistically be collected through ESM in a clinical setting (4 weeks of 5 measurements per day results in 140 measurements). Even when reducing the number of parameters through regularization, the number of observations per parameter remains low, indicating that power is likely suboptimal.

5 A contemporaneous association in a PSN can be validly interpreted as “when the patient reported A at a measurement moment, he typically also reported B at that moment.” A temporal association can be interpreted as “when the patient reported A at a measurement moment, he typically reported B at the next one.”

6 Note that these secondary benefits could be harnessed without using PSNs in psychotherapy.
