# Supplementary material for: Using person-specific networks in psychotherapy: challenges, limitations, and how we could use them anyway
Source: BMC Med. 2020 Nov 23;18:345. doi: 10.1186/s12916-020-01818-0 (PMC7682008; doi:10.1186/s12916-020-01818-0)
Supplement: Supplementary file 1 — Additional file 1: Table S1. Procedure for creating person-specific networks in the Therap-i trial. [file 12916_2020_1818_MOESM1_ESM.docx]

**Additional file 1**

**Table S1** Procedure for creating person-specific networks in the Therap-i trial. The procedure here is intended as an example, not as a recommendation.

| Choices in data collection |  |
| --- | --- |
| What variables to include? | A fixed set of 20 single-item variables that were selected to capture essential factors in depression (e.g., rumination, self-worth) and daily life experiences (e.g., general affect, activities, physical discomfort, perception of social interactions, perception of daily events).  About 10 single-item variables that are selected for the individual patient in collaboration between the patient, their therapist, and a researcher. Special attention is paid to including variables that capture strengths of the patient. Patients are allowed to add items at later stages. |
| How many variables to include? | Of the about 30 items assessed, about 20 items provide continuous data from visual analogue scales. These are considered for the PSN, but some may be excluded on the basis of the assumption checks described below. |
| How to measure included variables? | ESM protocol with 5 assessments per day at fixed intervals for a period of 8 weeks. Assessments are 3 hours apart. Patients receive a link via text message that can be used to fill in the questionnaire in the browser of their mobile device.  Feedback on PSNs is provided twice during the 8 weeks. Each PSN is based on 4 weeks of monitoring (up to 140 measurements).  ESM questionnaires include momentary (“At this moment…”) and retrospective items (“Since the previous beep…”). Personalized items are either chosen from an item repository (includes 91 items) or formulated for the individual patient. |
| Choices in modelling |  |
| Pre-processing | If a variable exhibits a significant trend of time in a linear regression, it is detrended by using the residuals from this regression as data input for the PSN. |
| Checking Assumptions | Variables are excluded from the PSN on the basis of the following criteria:   - less than 80% of measurements completed (also excludes variables that were not assessed over the full 4 weeks) - multicollinearity, as indicated by the variance inflation factor - lack of variation, as indicated by the inter-quartile range - non-stationarity, as indicated by the Kwiatkowski-Phillips-Schmidt-Shin test - non-normality, as indicated by the Jarque-Bera test |
| Modelling | Network models are estimated using the R-package graphicalVAR, which estimates temporal associations in a regularized vector-autoregression and contemporaneous associations in a regularized Gaussian graphical model based on the residuals of the former. Parameters in the estimation procedure: gamma = 0, nLambda = 50 |
| Choices in visualization |  |
| Network graphs | Network graphs are created using the R package qgraph. Node placement is based on the averageLayout function (repulsion = .85). The threshold parameter, which excludes connections below the threshold, can be set dynamically using a Shiny app. |

Note: PSN, Person-Specific Networks; ESM, Experience sampling method
